# Researching the Experiences of Children with Cancer: Considerations for Practice

**DOI:** 10.3390/children6080093

**Published:** 2019-08-16

**Authors:** Jessika Boles, Sarah Daniels

**Affiliations:** 1 Child Life Services, Monroe Carell Jr. Children’s Hospital at Vanderbilt, Nashville, TN 37232, USA; 2 Child Life Program, St. Jude Children’s Research Hospital, Memphis, TN 38163, USA

**Keywords:** pediatric oncology, healthcare research, research methods, children, adolescents

## Abstract

Children and adolescents with cancer often participate in medical and psychosocial research throughout their diagnosis and treatment. Furthermore, this involvement frequently extends into the survivorship period. Sometimes referred to as “doubly vulnerable” research participants, children and adolescents with cancer are not only minors, but also minors facing significant medical, developmental, and psychosocial stressors associated with chronic illness. Thus, it is important to exercise care in designing and conducting research with this population; however, these considerations have not been adequately addressed in pediatric healthcare literature. Therefore, the purpose of this review is to describe the research preferences and experiences of children and adolescents with cancer to identify techniques for supporting this population as research participants. By incorporating developmentally appropriate, context-specific, and child-centered adjustments, researchers can help children and adolescents with cancer effectively and meaningfully describe their illness experiences while also developing a positive outlook on future research participation.

## 1. Introduction

More than 13,000 children are expected to be diagnosed with cancer in the United States in 2019, more than eighty percent of whom will achieve long term survival [1]. Some of these improvements in cancer care and survival rates can be attributed to advances in medical technology and training; however, many are the direct result of child and adolescent participation in clinical trials and adjuvant research [2]. In fact, according to Unger and colleagues [3], it is estimated that more than 50 percent of children with cancer under the age of 15 agree to participate in clinical trials—a proportion ten times greater than what is found for adults with cancer, perhaps in part due to initiatives set forth by the Children’s Oncology Group (COG). Children and adolescents with cancer have cited a multitude of reasons for participation in research, though most frequently due to hopes for personal benefit (medical, psychosocial, or financial) and desire to help others (improving treatments or illness management, or to help parents and clinicians out of a sense of gratitude) [4].

In addition to clinical trials, many children and adolescents with cancer participate in social sciences research during treatment [5]. Such studies help to inform psychosocial support, educational interventions, and healthcare providers’ developmental understandings of children’s cancer treatment experiences. These lines of research may not involve invasive medical procedures or unfamiliar medical encounters; however, social sciences research also imposes burdens on the child and family’s time, energy, coping, and development [6]. The potential benefits of these studies can outweigh the minimal risks of participation, as outcomes improve providers’ understandings of the immediate and long-term impacts of cancer treatment on the child’s development, coping skills, and even the mental and physical health outcomes children may achieve across the lifespan [7]. In addition, these studies have broadened the perspectives of the medical to consider the ways in which childhood cancer and its treatment impacts not only the child, but also his or her family, community, and healthcare providers.

Knowing the multiple and far-reaching medical and psychosocial effects of childhood cancer, and the frequency with which patients and families must balance concurrent cancer treatment and research participation, it is important to carefully consider the vulnerabilities of this patient/participant population. Therefore, all types of research with children and adolescents with cancer should weigh concern for the child’s medical and psychosocial wellbeing with developmentally appropriate opportunities for children to describe their experiences, identify their needs, and express themselves while acting as participants. In addition, given the likelihood of children with cancer to be approached for research participation throughout their lifetime, it is essential to note that each participation opportunity “may shape their experiences of research participation, compliance with research procedures, and willingness to participate in future studies” [8] (p. 88). Therefore, the purpose of this paper is to describe current findings on children and adolescents’ experiences of participating in research, and identify considerations for developmentally appropriate, context-sensitive, and child-centered research design and conduct in pediatric oncology.

## 2. Children with Cancer: Illness Experiences

It has long been recognized that childhood cancer imparts a host of developmental and psychosocial impacts on individual, family, and community levels. Regardless of the child’s age and spectrum of ability prior to diagnosis, frequent hospitalizations, intensive multi-modal treatment protocols, and the social-emotional burden of the illness experience jeopardize, slow, regress, or accelerate the child’s developmental trajectory [9]. For instance, subgroups of survivors of childhood cancer have demonstrated delayed or decreased achievement of milestones in social development, such as initiating and maintaining romantic relationships and building a family [10]. In addition, survivors are more likely to require special education services, and less likely to graduate high school than the general population [11]. Furthermore, even when diagnosed and treated in the adolescent years, cancer survivors report higher rates of depression, anxiety, and neuro-cognitive impairment related to memory, task analysis, and self-regulation—many of which correlate with increased unemployment rates in the adult years [12].

Childhood cancer treatment not only impacts physical, psychological, and social development, but also limits a child’s participation in community contexts such as school [13]. More specifically, children with cancer have reported feelings of loneliness, confusion, and “being different” when separated from their school environment during treatment [7]. While school participation is only one example of the psychosocial impact of childhood cancer, it is a significant one because of the social meaning and importance that children assign to it [14]. Furthermore, children’s school experiences—whether in the community, at home, or in the hospital—are their primary vehicle for social relationships with peers, non-familial supportive adults such as teachers and staff, and opportunities to practice both cognitive and social skills [15].

Just as the child must navigate changes in his or her social relationships, family roles and responsibilities also shift to meet the demands of hospitalizations, therapies, and side effects related to pediatric cancer. Depending on the supports and resources available, and the family’s prior experiences, these shifts may be accomplished with anywhere from minor to severe disruptions in family functioning [16]. During this time of stress and adjustment, parents of children with cancer are also expected to play a key role in decision-making related to treatment, procedures, and research participation [17]. Thus, the family is also subject to the challenges and stressors imparted by a childhood cancer diagnosis; simultaneously, it is the child’s family that greatly shapes the medical, psychosocial, and developmental outcomes of his or her illness experience.

Treatment decision-making is a well-researched component of pediatric oncology; however, decision-making in the context of research participation is not as well understood. Studies have shown that young children are able to understand the integral aspects of research participation [18] and, as would be expected, this understanding grows more complex with age and continued development [19]. Despite this increasing ability to comprehend research enrollment and participation, a recent review found that only nine of 52 studies on randomized clinical trial enrollment reported patient preferences alongside those of parents [20]. As multiple authors and agencies have asserted, “children should not be either burdened or excluded from participation in research” [18,20,21,22]. Legally, children under the age of 18 years cannot consent to treatment unless there are specific extenuating circumstances (i.e., the adolescent is a legally emancipated minor) [23]; however, children under the age of 18 should be asked to provide assent—especially for research procedures that do not offer direct benefits to the child [24]. As these decisions are often made by weighing the potential risks and benefits of participation [21], and participation directly involves and impacts the child or adolescent, it is essential that providers and researchers utilize age-appropriate, context-specific, and child-centered techniques for recruitment, informed consent/assent, and data collection as discussed in more detail below [14].

## 3. Children with Cancer: Research Experiences

Compared to children with other chronic illnesses, children and youth with cancer must consider information about research at a time when they are likely also experiencing intense physical discomfort and disruption to their normal lives [25,26]. Consents are required prior to beginning therapy and are also required for various procedures that occur throughout treatment. It is plausible that the number of consent documents, let alone the need to understand these documents, is very overwhelming for parents and children [23]. The stress associated with diagnosis may impact a child’s ability to understand the consent process [27,28], and this level of stress may persist past the days of diagnosis into the treatment adjustment period [29,30]. For others, however, this stress may start to subside as the diagnosis is named, treatment is planned, and care becomes more routinized and familiar [29]. With regards to the timing of research enrollment, it has been found that children who provide informed consent at least one week following diagnosis report a higher understanding of their research participation than those who consent closer to the time of diagnosis [31]. At times, however, the urgency to obtain consent for treatment may inhibit the provision of dedicated time for the child to fully comprehend their assent. Indeed, when asked about enrollment in clinical research trials, one study revealed that 86% of children did not understand their doctor when the trial was discussed and 38% did not feel they had a choice to dissent to enrollment [32]. Given the gravity of these research participation decisions, it is legally and ethically indispensable that children are given the resources they need to comprehend and issue their informed decision, rather than simply relying upon “non-refusal” of participation [23].

Aside from clinical trials focusing on disease management, children and adolescents with cancer frequently participate in research studies driven by other medical and psychosocial disciplines [33,34]. Epidemiology, psychology, biology, and genetics are just a few areas researching the unique experiences of children with cancer [35]. Other areas of study for this population include parenting concerns [36], cognitive and neuropsychological functioning [37], and patient experiences within and outside of the hospital [38]. Both social science and clinical research traditions acknowledge the challenges in striking a balance between protecting the vulnerabilities of young research participants and respecting their autonomy [5,33]. However, it is rare for these disciplines to communicate with one another about the best approaches for achieving this balance [5]. In addition, across fields of research, there remain significant racial, ethnic, and socioeconomic disparities in children’s access to and involvement in healthcare research [39]; these disparities potentially reinforce the already existent inequities in the provision of healthcare in general [40].

The methodologies used with children with cancer in clinical and psychosocial research can vary greatly across study objectives, theoretical perspectives, or feasibility when resources are constrained. The most revered study designs in medical research are typically randomized controlled trials, followed by meta-analyses, systematic reviews, and rigorous experimental or observational designs [41]. However, pediatric oncology research can also be qualitative or mixed-methods in design. Since quantitative and qualitative research are typically grounded in different epistemologies, or understandings of knowledge, they will often employ different methods for collecting data from participants [42].

A common quantitative data collection technique used in research interactions with children with cancer is the administration of written or verbally proctored questionnaires [43]. In comparison, qualitative studies often rely upon ethnographic, observational, and interview-driven techniques to capture data in real time [42]. Other qualitative approaches used include the draw-to-write method, interview-elicited drawings, arts-based activities, or participatory visual methods [14,44,45]. These methodological selections may be derived from the researcher’s academic training, professional role, or personal experiences, preferably with consideration to the developmental needs and capabilities of the intended participant population. Regardless of the research methods implemented, both social sciences and clinical researchers must consider the influence of development on children’s interest in, and ability to participate meaningfully in, research about their experiences [5]. Unfortunately, and more difficult to avoid, is that, even when child-centered data collection methods are used, the interpretation of research findings involving children is almost always framed from an adult researcher’s perspective [46].

Understanding children’s views on participating in research is the first step to presenting their perceptions accurately and thus generating more informative and useful research findings. To date, only a few articles exist that report on children’s attitudes and beliefs about participation in research during cancer therapy. Of these articles, children have reported several motivations for participating in healthcare research, including direct health benefits, altruism, and hope for an improved quality of life [43,47]. Simultaneously, children with cancer are acutely aware of the risks of research participation and have cited concerns about potential side effects and prolonged hospitalization when making enrollment decisions [48,49], unenjoyable or painful tasks required as part of participation [19], and desires to avoid further disruption to daily life [43]. For instance, Hunfeld and Passchier [19] found that children are more likely to participate in studies that do not involve painful data collection techniques; other studies have shown that older adolescents, conversely, are more willing to undergo additional potentially uncomfortable procedures for research purposes if it means they would be helping others [50]. Regardless of age and developmental level, all children and adolescents are more likely to decline participation in studies that could extend their separation from home and peers [49].

In addition to considering the child’s perspectives on research participation, it is also important to learn about the child’s understanding of their involvement in research decision-making. Even today, several decades after the United Nations’ Convention on the Rights of the Child (UNCRC) [51], children’s informed consent remains tenuous depending on the type of study in which he or she will enroll, and the presence and preferences of legal guardians (parents or otherwise) [52]. Notably, “informed consent” is difficult to quantify, especially when assessing a child’s understanding of his or her potential involvement in research—and the risks and benefits associated with such participation [18]. Thus, the concept of “assent” is often considered enough for research enrollment, especially when given alongside a parent or legal guardian’s informed consent [23]. However, there are many practical, legal, and ethical problems surrounding children’s assent, which has been poorly operationalized or standardized in healthcare research; thus, it should be practiced that “children’s failure to dissent [the capacity and opportunity to ‘say or express no’] does not necessarily signal their voluntary consent…not all children want to participate in research and not all know how to say or express ‘no’” [52] (p. 152).

When presented with age-appropriate information in a supportive context, children and adolescents with cancer can understand the complex components of cancer treatment and research participation. At the same time, some studies regarding what children with cancer understand about clinical trial research conclude that some children may have limited understanding and are confused about the difference between research and treatment [27,32]. More specifically, children demonstrate a higher understanding about the objectives, risks, and benefits of research participation, but are less understanding of the procedures and duration of studies, the voluntary nature of study participation, and the possibility of alternative treatments [19,31]. A study reviewing the informed consent process with children with cancer found significant variance among clinician communication with children when parents are present [53]. During informed consent conferences, only 7% of dialogue was directed toward children as a result of their asking questions. Even then, children’s questions most often concerned disease and treatment-related questions as opposed to questions about the research study [53], validating that there are multiple areas for growth in research enrollment and communication strategies in this population.

Children and adolescents with cancer are expressive regarding their motivations and perceived hindrances for engaging in clinical research [19,48,49]. These views are reflective of their ability to understand the complexities in decision-making about research participation, even in the context of the stress associated with cancer care. However, children may still have some misunderstandings about research in the hospital setting, namely when treatment and research are intertwined [32]. Therefore, beyond requiring child assent to participate, providers need to demonstrate respect for pediatric participants by enhancing communication about the proposed research, and by thoughtfully designing age-appropriate studies.

## 4. Considerations for Research Practices

Independent of research objectives, researcher training, and study design, modifications can be made to best support the developmental and psychosocial needs of children and adolescents with cancer participating in research. Such adjustments can not only improve the quality, quantity, and accuracy of data collected, but also promote children’s interest in continued research participation during and beyond their cancer treatment [8,54].

Based on the literature currently available, it is essential that researchers and clinicians consider opportunities for (1) improving the informed consent or assent processes, (2) attending to relationships from study enrollment through completion, (3) offering participants choices and control in the context of research participation, (4) incorporating flexible research designs and expectations, and (5) utilizing developmentally appropriate, varied, and enjoyable data collection methods.

### 4.1. Improving the Informed Consent Process

It is important for all providers to remember that “the research context may be unlike any experiences children and youth have had” [54] (p. 508). With an intentionality towards and awareness of the child’s perspectives, providers are equipped to adapt the informed consent process in a way that enhances a child’s understanding through clearer distinctions about research-related and treatment-related information [4]. Informed consent conferences have extremely low levels of physician-to-patient communication, but this type of communication improves child understanding about research studies. Physician-to-patient communication during informed consent conferences is also disproportionately higher during conferences with older adolescents [48]. Thus, enhancing child understanding requires increased physician-to-patient communication with children of all ages. This means that providers should communicate directly with the child or adolescent, rather than speaking only with the caregiver in informed consent conferences [4]. In addition to giving information, physician-to-patient communication should be socio-emotional, including statements of reassurance and validation, should involve asking and checking understanding, and should establish a partnership with the patient in decision-making related to the study [8,48].

Physicians may be more comfortable communicating with caregivers because they expect them to relay information to their child or because they anticipate that the caregiver will make the ultimate decision [47]. Unfortunately, many caregivers may also be overwhelmed and unaware of how to best discuss enrollment with their child. This is especially true when there are higher stakes for trial enrollment, such as when standard treatments have not worked for their child [48]. When explaining the child’s research participation and role, it may be helpful for physicians to call on psychosocial providers, such as Certified Child Life Specialists, to be present for these conversations. Certified Child Life Specialists are experts in child development and may be especially helpful in recommending age-appropriate communication strategies for parents or providers [55].

### 4.2. Developing and Maintaining Relationships with Participants

Numerous studies have shown that children and adolescents with cancer expect and value the relationships they build with staff while participating in research [5,8,18,56]. In fact, children and adolescents have also reported increased interest in research participation when they feel that their assent is valued, their opinions are sought, and their autonomy is preserved by research team members [8]. There are a variety of strategies by which to build rapport with child and adolescent participants, depending on the child’s developmental level, communication preferences, and personal interests; regardless of individual differences, it is important to attend to both what children do/say and what they do not do/say as a means of recognizing the verbal and nonverbal communications necessary for effective relationship building [52,57]. These relationships can be precarious, especially when children are participating in research and treatment simultaneously; however, with clear explanations, active listening skills, and the considerations below, researchers can build relationships with participants that are mutually beneficial and empirically invaluable [5].

### 4.3. Offering Choice and Control in the Context of Research Participation

Providing the child or adolescent with increased choice and control over assent and research processes may also improve understanding and participation. For example, using multimodal communication opportunities, such as drawings and artistic expressions, along with conversation can encourage children to share their views or questions in a way that is comfortable for them [46]. It is also recommended that physicians provide children or adolescents with their own information and consent forms [58]. One way to empower the child research participant is by providing them with a choice of how they wish to give assent. For example, some children may feel more comfortable verbally providing assent, whereas others may wish to physically sign their consent form. Giving a signature, just as their caregiver does, can empower some youth in their decision-making [47].

For adolescents with cancer who are already missing out on other developmentally appropriate milestones marking independence, such as getting their driver’s license or moving away to college, giving them ample attention and opportunity to recognize their role in research is one way to honor their autonomy [59]. One suggestion for providing adolescent research participants with increased control in decision-making is to offer a moment for the adolescent to speak with the physician without a caregiver present [48]. Adolescents report feeling pressured to make decisions based on what their parents would recommend [60]. Giving the adolescent time away from their parents will provide them with space to consider their own thoughts related to the study.

### 4.4. Incorporating Flexible Research Designs and Expectations

Research practices with children with cancer may also be improved by anticipating scheduling barriers and enhancing study flexibility, when appropriate, to accommodate treatment and normalization needs [5]. Introducing many studies right at the time of diagnosis can contribute to already heightened anxiety and stress felt by the child and family [27]. Any study that can wait to enroll patients, even just one week after diagnosis, may provide the child and family time to adjust to diagnosis and attend to the research discussion. Children and adolescents with cancer have reported that extended separation from home and peers is a major deterrent to their research enrollment [49]. Cancer patients already endure various appointments and procedures throughout each day at the hospital. Additional appointments scheduled solely for research purposes may not be necessary, and fewer hospital visits may enhance research participation.

Some institutions have advisory boards of child and adolescent participants who can share perspectives about which experiences are burdensome in research [61]. Advisory groups like these can help to raise awareness about what expectations are reasonable for child research participants. One consideration to make research design more flexible is to consider modifying follow up conversations about study progress through digital outlets, such as phone calls or online platforms [61]. By listening to children’s perspectives on hindrances to participating in research, providers may be able to alter their study designs in ways that accommodate the child’s needs, thus improving adherence to studies overall [20].

### 4.5. Utilizing Developmentally Appropriate and Enjoyable Data Collection Methods

So as not to add additional stress to the patient and family, the design and methods of a research study involving children and adolescent participants should include flexible and enjoyable procedures. The researcher should establish rapport with the child in a non-threatening environment to build a trusting relationship prior to conducting research [5,44]. Additionally, it may be appropriate to utilize technology or play to enhance engagement and understanding of study rationale [5,19]. It may also be feasible in some instances to include children in the research design process by seeking their input in the development of questions that they can understand [62].

All children are different, and many children will disagree about their preferred method for giving information. This means that no single research method is most appropriate to use when collecting data from children. Some children will enjoy arts-based tasks whereas others will find story-telling to be enjoyable [63]. Research methods that are common in adult research, such as interviews and focus groups, may also be appropriate for research with children. However, researchers will need to consider child and illness variables, such as attention span and disease status, in order to enhance research of children’s experiences [63]. Finally, after researchers collect data using child-centered methods, they must also remember to present their findings with respect to the child perspective. Utilizing developmentally appropriate and enjoyable methods of data collection are important steps for engaging the child in meaningful research [5].

## 5. Discussion

Compared to their healthy peers, children with cancer have many unique opportunities to participate in research studies throughout their lifetime [7]. Without child and family participation in research throughout cancer treatment, advancements in care would not be possible. The medical and psychosocial communities rely on patient and family research participation to inform changes in practice. However, research participation can be stressful and confusing for children and families who are already enduring the stress of a cancer diagnosis. To protect patients from additional stress, providers should introduce research opportunities in ways that promote a positive outlook on research participation over time.

A main goal of clinical trial research is to evaluate generalizable trends and impacts of treatments on cancer outcomes. This aim inherently calls for quantitative study designs that randomize participants, recruit large sample sizes, and require strict adherence to the scheduled treatment plan. There are child and adolescent assent requirements in place to ensure that patients understand and agree to participate in clinical research. While these practices intend to educate patients about their rights related to research participation, it is evident that many children and adolescents feel pressured to assent, either by their parents’ or their own concerns about delaying the start of treatment. Moreover, while the assent process may be helpful in delegating some sense of control to children and adolescents with cancer, this one act alone is not sufficient in making research a valuable experience for children and adolescents with cancer.

In the limited literature describing the perspectives of children and adolescents with cancer about research, it is clear that they understand the complexities of decision-making regarding research participation. What is less clear is whether this understanding is heard and respected among providers. Children with cancer are motivated to engage in research activities for some purposes, like when they feel it will directly impact their health status [47], but they are less aware of the existence of alternative treatments. Additional factors, including the invasiveness and the duration of a research study, may impact a child’s willingness to participate in research [19].

Clinical trial research is meaningful and necessary for advancement in cancer treatment, but children and adolescents with cancer should also be given opportunities to engage in research that meaningfully represents their perspectives and experiences. As articulated by Mack, Giarelli, and Bernhardt [64], engaging children and adolescents in research "requires flexibility, constant vigilance to cues from the participant, and a genuine interest in the feelings and ideas of this cohort. The primary tasks for the research interviewer are to build rapport, guide the pace of responses, and assure that information is complete and accurate while ensuring that all the rights of the participant are respected. The most effective research interviewers apply developmental concepts to the task, share power, accept unconditionally, and continually evaluate rapport" (p. 451).

Involving children and adolescents in research in this manner can transform the research participation experience from one that is stressful and uncertain into one that may be a positive, productive, and therapeutic developmental experience as the child is given time and space to voice their perceptions and needs [57].

Without a clear representation of child perspective and experience throughout cancer treatment, medical and psychosocial providers risk misunderstanding data and adapting practices in ways that are not appealing to their patients. Engaging children and adolescents in research that uses flexible and enjoyable methodology is one way to create a positive impression of research participation while also representing the child and adolescent voice. If providers are cognizant of children’s motivations and preferences, they may enhance their communication from the outset and thereby ensure more ethically sound and developmentally appropriate research initiatives.

## 6. Conclusions

In the grand scheme of oncology research, trials and studies with children and adolescents receive less funding and support than those enrolling adult patients [65]. Despite this lack of resourcing and the multitude of stressors for children and families undergoing cancer treatment, children and adolescents with cancer are interested in and capable of participating in research for a variety of reasons. The burden of responsibility, then, is on the research community to understand the developmental needs and preferences of children and adolescents with cancer, as well as their research participation motivations and turn-offs, to ensure that research designs are developmentally, psychosocially, and ethically tailored to this uniquely vulnerable participant group.
